# Supplementary material for: Coupling of saccade plans to endogenous attention during urgent choices
Source: bioRxiv. 2024 Mar 15:2024.03.01.583058. Preprint. [Version 2] doi: 10.1101/2024.03.01.583058 (PMC10942325; doi:10.1101/2024.03.01.583058)
Supplement: Supplementary file 1 [file NIHPP2024.03.01.583058V2-supplement-1.pdf]

bioRxiv preprint doi: https://doi.org/10.1101/2024.03.01.583058; this version posted March 15, 2024. The copyright holder for this preprint (which was not certified by peer review) is the author/funder, who has granted bioRxiv a license to display the preprint in perpetuity. It is made available under aCC-BY 4.0 International license.

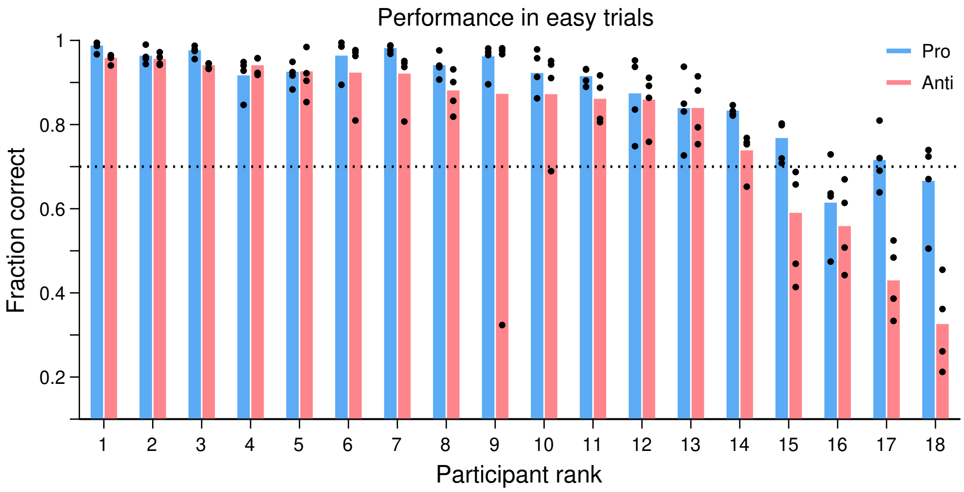

Figure S1. Performance in easy trials and inclusion criterion. Easy trials were those in which the cue and non-cue stimuli were revealed before the go signal was given (gap < 0), so participants typically had more time to process the cue information. Bars show mean performance in easy pro (blue) and easy anti trials (red), averaged across experiments, for each participant. Black dots indicate performance in easy trials in each of the four experiments. The performance criterion was met when the fraction correct in easy trials was above 0.7 (dotted line). Participants who met the criterion in both tasks (pro and anti) in all four experiments were considered reliable performers ($n = 11$; participants ranked 1–8 and 11–13). Others ($n = 7$) were considered unreliable performers. Participants are sorted by their overall antisaccade performance.

bioRxiv preprint doi: https://doi.org/10.1101/2024.03.01.583058; this version posted March 15, 2024. The copyright holder for this preprint (which was not certified by peer review) is the author/funder, who has granted bioRxiv a license to display the preprint in perpetuity. It is made available under aCC-BY 4.0 International license.

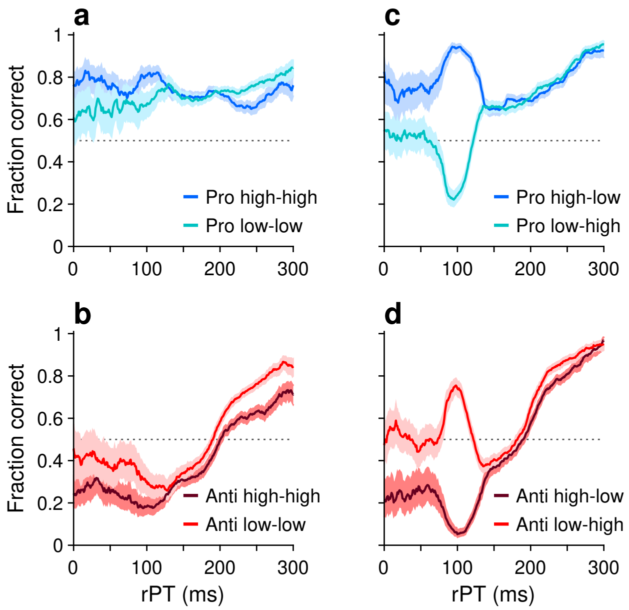

Figure S2. Tachometric curves from unreliable performers. The plots in this figure are analogous to those in Fig. 2, but present data from the participants that did not meet the performance criterion ($n = 7$). (**a**) Tachometric curves for pro trials in Experiments 1 (high luminance cue, high luminance non-cue) and 2 (low luminance cue, low luminance non-cue). (**b**) As in **a**, but for anti trials. (**c**) Tachometric curves for pro trials in Experiments 3 (high luminance cue low luminance non-cue) and 4 (low luminance cue, high luminance, non-cue). (**d**) As in **c**, but for anti trials. Luminance combinations for cue and non-cue are indicated for each curve. Error bands indicate 95% CIs across trials. During guesses (rPT $\leq$ 75 ms), the saccades of unreliable performers are strongly biased toward the cue, but additional exogenous capture is clearly visible in Experiments 3 and 4.

bioRxiv preprint doi: https://doi.org/10.1101/2024.03.01.583058; this version posted March 15, 2024. The copyright holder for this preprint (which was not certified by peer review) is the author/funder, who has granted bioRxiv a license to display the preprint in perpetuity. It is made available under aCC-BY 4.0 International license.

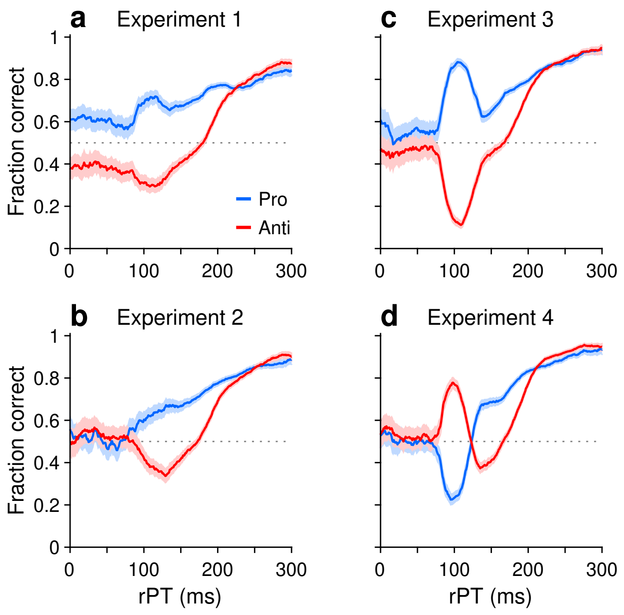

Figure S3. Prosaccade versus antisaccade performance aggregated across all the participants. Each panel compares the tachometric curve for pro trials (blue) with that from anti trials (red) based on data from a given experiment pooled across all the participants. (**a**–**b**) Tachometric curves from Experiments 1–4, as labeled. Error bands indicate 95% CIs across trials. In all cases, the endogenously driven rise in performance (for rPT $\gtrsim$ 135 ms) occurs earlier during pro trials than during anti trials.

bioRxiv preprint doi: https://doi.org/10.1101/2024.03.01.583058; this version posted March 15, 2024. The copyright holder for this preprint (which was not certified by peer review) is the author/funder, who has granted bioRxiv a license to display the preprint in perpetuity. It is made available under aCC-BY 4.0 International license.

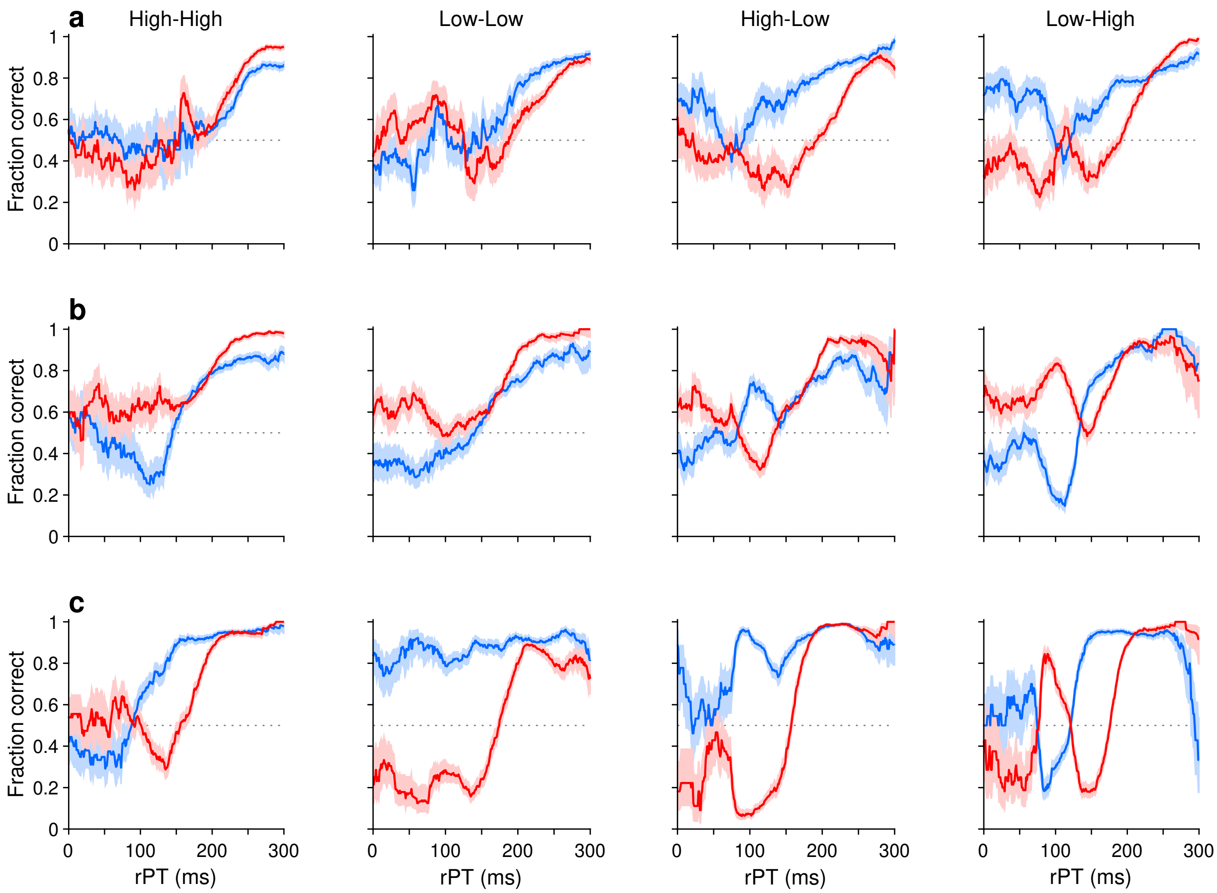

Figure S4. Individual participants demonstrate varying degrees of bias and exogenous capture. Each panel shows two tachometric curves, one for prosaccades (blue) and another for antisaccades (red). Columns 1–4 correspond to Experiments 1–4, as indicated. Shaded error bands indicate 68% CIs. (**a**) Data from a participant demonstrating a slight bias toward the cue (columns 3, 4) and minimal exogenous capture. (**b**) Data from a participant demonstrating a moderate, consistent bias toward the non-cue and a moderate level of exogenous capture. (**c**) Data from a participant demonstrating a sometimes strong bias toward the cue (column 2) and strong exogenous capture.
